# DCMA: faster protein backbone dihedral angle prediction using a dilated convolutional attention-based neural network

**DOI:** 10.3389/fbinf.2024.1477909

**Published:** 2024-10-18

**Authors:** Buzhong Zhang, Meili Zheng, Yuzhou Zhang, Lijun Quan

**Affiliations:** ^1^ School of Computer and Information, Anqing Normal University, Anqing, China; ^2^ Jiangsu Provincial Key Laboratory for Computer Information Processing Technology, Soochow University, Suzhou, China; ^3^ School of Information Engineering, Nanjing Xiaozhuang University, Nanjing, China; ^4^ School of Computer Science and Technology, Soochow University, Suzhou, China

**Keywords:** protein dihedral angles, lightweight model, dilated convolution, multi-head attention, hybrid inception blocks

## Abstract

The dihedral angle of the protein backbone can describe the main structure of the protein, which is of great significance for determining the protein structure. Many computational methods have been proposed to predict this critically important protein structure, including deep learning. However, these heavyweight methods require more computational resources, and the training time becomes intolerable. In this article, we introduce a novel lightweight method, named dilated convolution and multi-head attention (DCMA), that predicts protein backbone torsion dihedral angles (ϕ,ψ) . DCMA is stacked by five layers of two hybrid inception blocks and one multi-head attention block (I2A1) module. The hybrid inception blocks consisting of multi-scale convolutional neural networks and dilated convolutional neural networks are designed for capturing local and long-range sequence-based features. The multi-head attention block supplementally strengthens this operation. The proposed DCMA is validated on public critical assessment of protein structure prediction (CASP) benchmark datasets. Experimental results show that DCMA obtains better or comparable generalization performance. Compared to best-so-far methods, which are mostly ensemble models and constructed of recurrent neural networks, DCMA is an individual model that is more lightweight and has a shorter training time. The proposed model could be applied as an alternative method for predicting other protein structural features.

## 1 Introduction

Proteins play important roles in biological activities and often fold into unique three-dimensional structures to perform their biological functions. However, experimentally determining protein tertiary structures is costly and time consuming. Predicting protein tertiary structures from their corresponding sequences is still a challenging problem in computational biology. An integral part of predicting tertiary structures is to predict interval structural properties, such as secondary structures, solvent-accessible surface area, backbone dihedral angles, and contact maps. The backbone structure of a protein can be described continuously by backbone dihedral angles ϕ and ψ . The backbone torsion angle prediction is beneficial for protein structure prediction. The dihedral angle prediction has many applications in protein structure prediction, including (i) better secondary structure prediction, (ii) generation of multiple sequence alignments, (iii) identification of protein folds, and (iv) fragment-free tertiary structure prediction (Singh et al., 2014). Many computational methods, especially deep learning-based models, have been applied in this field.

In 1993, a discrete approach was used to predict backbone dihedral angles that removed any approximations, including the assumption that the effects of adjacent residues were uncorrelated (Kang et al., 1993). In 2005, a continuous neural network-based method was proposed to predict protein secondary structure and backbone dihedral angles (Wood and Hirst, 2005). Other machine learning methods have also been applied to the prediction of protein dihedral angles, such as ANGLOR (Wu and Zhang, 2008) and TANGLE (Song et al., 2012) using support vector machines and neural networks, TALOS+ (Shen et al., 2009), SPINE X, and Real-SPINE3.0 using neural networks, DANGLE (Cheung et al., 2010) using Bayesian, conditional random field (Zhang et al., 2013), and so on.

In recent years, deep learning methods have been successfully applied to the prediction of protein structural properties, including protein backbone dihedral angles. A deep recurrent restricted Boltzmann machine (DReRBM) was developed to research protein dihedral angles (Li et al., 2017). RaptorX-angle combines K-means clustering and deep learning techniques to predict dihedral angles (Gao et al., 2018). Spider 3 (Heffernan et al., 2017), which eliminates the effect of the sliding window, used the machine learning model of the bidirectional long short-term memory (BLSTM) (Hochreiter and Schmidhuber, 1997) recurrent neural network (Schuster and Paliwal, 1997; Graves et al., 2014). The DeepRIN (Fang et al., 2018b) was designed based on the combination of the inception (Szegedy et al., 2016) and the ResNet (He et al., 2016) networks. SPOT-1D used an ensemble of BLSTM and ResNet to improve the prediction of protein secondary structure, backbone dihedral angles, solvent accessibility, etc. (Hanson et al., 2019). SPOT-1D integrated three LSTM models, three LSTMResNet models, and three ResNet-LSTM models and integrated contact maps as model input and to boost its performance. Klausen and colleagues proposed the NetSurfP-2.0 model, which used an architecture consisting of a convolutional neural network (CNN) and LSTM Networks (Klausen et al., 2019). Xu and colleagues proposed OPUS-TASS, a protein backbone dihedral angles and secondary structure predictor (Xu et al., 2020). It is an ensemble model; its individual model parts consist of CNN, LSTM, and modified transformer networks. Zhang and colleagues proposed CRRNN2, which introduced a multi-task deep learning method based on BRNNs, one-dimensional (1D) CNN, and an inception network, which can concurrently predict protein secondary structure, solvent accessibility, and backbone dihedral angles (Zhang et al., 2021). As an upgraded version of OPUS-TASS, OPUS-TASS2 integrated global structure information generated by trRosetta (Du et al., 2021) and achieves SOTA performance. OPUS-TASS2 adopts an ensemble strategy as OPUS-TASS and SPOT-1D, and it consists of nine models (Xu et al., 2022).

Recently, AlphaFold2 has achieved great success in predicting protein monomer structures (Jumper et al., 2021; Ismi et al., 2022). However, accuracy for single-sequence-based prediction of secondary structures is far from the theoretical limit of 86%–90% (Zhou et al., 2023). The bottleneck resides in the immense computational demands of running the AlphaFold2 model, both in terms of computing power and runtime. Therefore, there is still a need for prediction tools that can predict protein backbone angles in a faster and more accurate manner. A recurrent neural network (RNN) maintains a vector of activations for each timestep that can remember prior input to influence the current input and output. RNNs can be easily used for sequential or time series data (Jozefowicz et al., 2015). The best-so-far deep learning methods are essentially constructed by RNNs like SPOT-1D (Hanson et al., 2019), OPUS-TASS (Xu et al., 2020), OPUS-TASS2 (Xu et al., 2022), NetSurfP-2.0 (Klausen et al., 2019), and CRRNN2 (Zhang et al., 2021). Because the computation of each step in an RNN depends on the previous step, the recurrent computations are less amenable to parallelization. In contrast to the models built by convolution or attention networks, RNN-based models need more training and running times. Moreover, these heavyweight models consume more computing resources, which is not conducive to training or inference.

In this article, we designed a new hybrid inception block consisting of 1D CNNs and dilated CNNs (Yu and Koltun, 2016). As an alternative to an RNN, a novel architecture with two hybrid inception blocks and one multi-head attention (Vaswani et al., 2017) block called an I2A1 module is intended to capture local and long-range features, which are comprised of two hybrid inception blocks and augmented by one multi-head attention network. The dilated convolution and multi-head attention (DCMA) novel protein backbone dihedral angle predictor is mainly constructed from I2A1 modules. Hence, we have made the following outstanding contributions: 1) proposed a faster method that can substitute for RNNs and offers comparable performance, and 2) provided a more lightweight tool for predicting dihedral angles that is more friendly to biological or medical researchers.

## 2 Materials and methods

### 2.1 Datasets

We used the same training (Hanson et al., 2019) and validation sets as SPOT-1D and OPUS-TASS 1/2 for a fair comparison with most state-of-the-art methods. The sequences were culled from the PISCES server (Wang et al., 2003) by SPOT-1D in February 2017, with the following constraints: resolution > 2.5 Å, R-free < 1, a sequence identity truncation rate of 25%, and the sequence length ≤ 700. Finally, the training set and validation set contain 10,029 proteins and 983 proteins, respectively.

To evaluate the performance of different methods, we performed the method on six public independent test sets: (1) The CASP12 dataset contains 40 proteins; (2) CASP13, which contains 32 proteins; (3) CASP-FM (56), collected by SAINT (Uddin et al., 2020), which contains 10 template free modeling (FM) targets from CASP13, 22 FM targets from CASP12, 16 FM targets from CASP11, and 8 FM targets from CASP10; (4) the CASP12-FM dataset, collected by Singh et al. (2021a), which contains 22 FM proteins from CASP12; (5) the CASP13-FM dataset, collected by Singh et al. (2021a), which contains 17 FM proteins from CASP13; and the (6) CASP14-FM dataset, collected by Xu et al. (2022), which contains 15 FM proteins from CASP14.

### 2.2 Input features

DCMA takes three groups of sequence-based features as input: a position-specific scoring matrix (PSSM) profile, a hidden Markov model (HMM) profile, and residues coding. As SPOT-1D reported, each 20-dimensional protein PSSM was generated by three iterations of PSI-BLAST (Altschul et al., 1997) against the UniRef90 sequence database updated in April 2018. The 30-dimensional HMM sequence profiles are re-generated by HHBlits (v3.1.0) (Steinegger et al., 2019) with default parameters based on the UniRef30 database updated in June 2020. Similarly to CRRNN’s schema (Zhang et al., 2018), a one-hot vector of residues coding is mapped to a 22-dimensional dense vector.

### 2.3 Outputs

To remove the effect of the angle’s periodicity, we employ a pair of sine and cosine values for each torsion angle as the output instead of directly predicting ψ or ϕ . As a result, there are four outputs: sin⁡ψ , cos⁡ψ , sin⁡ϕ , and cos⁡ϕ .The predicted angle α is defined as

$\alpha =arctan\left(\frac{\sin\alpha }{\cos\alpha }\right)$

The multi-task learning strategy of predicting protein structural properties concurrently has been proven to be effective by CRRNN2 (Zhang et al., 2021). We also adopted the same multi-task learning schema as CRRNN2, in which the auxiliary output during the training period is protein secondary structure (Q3 and Q8) and solvent accessibility. The loss function and respective output ratio in DCMA are the same as in CRRNN2.

### 2.4 DCMA model

**FIGURE 1:**
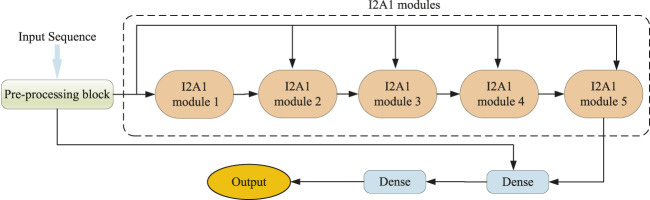
Model architecture of the DCMA.

**FIGURE 2:**
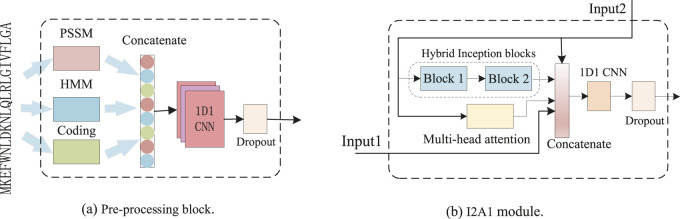
Architecture of pre-processing block **(A)** and I2A1 module **(B)**.

As illustrated in Figure 1, our DCMA model consists of three parts: a pre-processing block, five stacked I2A1 modules, and two fully connected layers. The input features are transformed in the pre-processing block, and its structure is demonstrated in Figure 2A. The I2A1 module is mainly constructed by two cascaded hybrid inception blocks and one multi-head attention block, as Figure 2B shows. The details of these blocks will be introduced below.

#### 2.4.1 Pre-processing block

As Figure 2A and Equation 1 show, the representing residue features, including 20-dimension (D) PSSM, 22-D residue coding, and 30-D HMM, are aggregated and transformed into 256-D tensors by one dimension and one kernel (1D1) CNN (Zhang et al., 2018). The weight constraint of dropout (p = 0.5) used to avoid overfitting was applied to the output of 1D1 CNN. Then, the tensors denoted as input are fed to each I2A1 module and the first dense layer.

$$\begin{array}{cc} con & =contenate\left(PSSM,HHM,Coding\right)\\ Output & =dropout\left(1D\text{\_}CNN\left(con\right),\;p=0.5\right). \end{array}$$ (1)

#### 2.4.2 Hybrid inception block

CNNs provide the property (Strubell et al., 2017) that parallelizes runtime independent of the sequence length maximizes GPU resource usage and minimizes the training and evaluating time. However, a CNN’s perception is limited by the input size. As Strubell’s description notes (Strubell et al., 2017), the maximum perception length r of CNN is expressed as r=l(w−1)+1 , where l is the stacked layers number, and w is filter size. And the receptive width is promoted to $2^{l+1}-1$ , when l dilated layers are stacked. Dilated convolution can cover a larger area of the input due to skipping some areas, and a large portion of the information is lost (Wang et al., 2018). A simple and useful solution is hybrid dilated convolutions, and this strategy has been proven practicable (Wang et al., 2018; Wu et al., 2016; Chen et al., 2017; Singh et al., 2021b).

**FIGURE 3:**
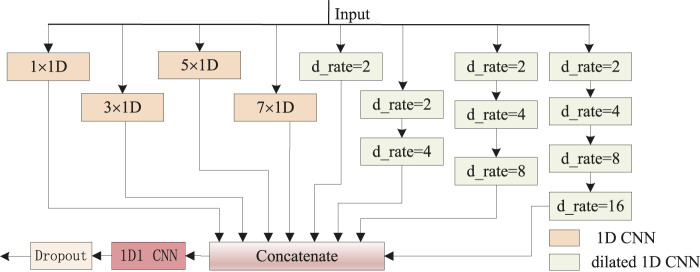
Newly hybrid inception block. The 1D CNNs with 64 filters and kernel size [1, 3, 5, 7] are intended to capture sequence local futures. Dilated 1D CNNs with 64 filters, kernel size 2, and multi-scale dilation rates (d_rate) are used to capture sequence long-range dependencies.

Motivated by the effectiveness of the inception (Szegedy et al., 2015; Szegedy et al., 2016) network based on CNNs (Fang et al., 2018b; Fang et al., 2018a; Uddin et al., 2020), we proposed a newly hybrid inception block, as shown in Figure 3. Four types of local features are aggregated from 1D CNNs with 64 filters and kernel size [1, 3, 5, 7] respectively, for the minimum length of protein secondary structures is three, and 1D CNN with kernel size one (1D1 CNN) is used to data dimension transformation. Another four groups of long-range features are perceived from hybrid dilation rates (d_rate) CNNs with 64 filters and kernel size 2. Similar to DeepLabv3’s (Chen et al., 2017) configuration, dilation rates [2, 4, 8, 16] are applied. For better perceptive capability, four channels with stacked [1, 2, 3, 4] dilated CNNs, respectively, are combined. Multi-scale dilation rates { [2], [2, 4], [2, 4, 8], [2, 4, 8, 16]} are used in corresponding channels, respectively. When the eight parallel outputs are concatenated as a 512-dimensional tensor, the data dimension is transformed into 256 by a 1D1 CNN for model weight reduction.

### 2.4.3 Multi-head attention

The attention mechanism (Tay et al., 2020) can be viewed as a graph-like inductive bias that connects all tokens in a sequence with a relevance-based pooling operation. Multi-head attention (Vaswani et al., 2017) allows the model to jointly attend to information from different representations and focus on different aspects of information. The advantage of attention is that it can capture long-term dependencies without being limited by sequence length. Because the result of each step does not depend on the previous step, steps can be done in parallel mode. We use a multi-head attention mechanism as a complement to the hybrid inception block. Eight heads are employed. In the DCMA model, we use multi-head attention as Equations 2–4, the most popular attention in recent years, which combines multiple self-attention networks to divide the model into multiple heads to form multiple subspaces and can make models focus on different aspects of information. In our model, we employ eight heads, and tanh activated function is added to the output for smooth variation. In order to control model weight, the dimension of ${\text{head}}_{i}$ is reduced to 64, and the output dimension of the attention block is 256.

$$Output=tanh\left(MultiHead\left(Q,K,V\right)\right),$$ (2)

$$MultiHead\left(Q,K,V\right)=contenate\left(head_{1},head_{2}\ldots head_{8}\right)W^{O},$$ (3)

$$head_{i}=Attention\left(QW_{i}^{Q},KW_{i}^{K},VW_{i}^{V}\right)==softmax\left(\frac{QW_{i}^{Q}{\left(KW_{i}^{K}\right)}^{T}}{\sqrt{d}}\right)VW_{i}^{V}.$$ (4)

#### 2.4.4 I2A1 module

The input data is fed parallel to one multi-headed attention block and two cascaded hybrid inception blocks, as Figure 2B and Equations 5–10 show. The proposed module can effectively capture both the short-range and long-range dependencies. In the first I2A1 module (*i* = 1), Input2 is the output of the pre_processing block, and Input1 is null. In other I2A1 modules, Input2 is the output of the previous I2A1 module, and Input1 is the output of the pre_processing block. For a better balance between the ability to model long-range dependencies and computational efficiency, only one attention block is joined with two cascaded hybrid inception blocks, and the dimension of concatenated data is also reduced from 1,024 (768, when in the first module) to 256 by 1D1 CNN.

$$Input1_{i}=\left\{\begin{array}{c} null,\;if\;\;i=1\;\\ output\;of\;pre\text{\_}processing\;block,\;if\;i>1,\; \end{array}\right.$$ (5)

$$Input2_{i}=\left\{\begin{array}{c} output\;of\;pre\text{\_}processing\;block,\;if\;\;i=1\;\\ output\;of\;previous\;I2A1module,\;if\;i>1,\; \end{array}\right.$$ (6)

$$F\text{\_}out1_{i}=inception\left(inception\left(Input2_{i}\right)\right),$$ (7)

$$F\text{\_}out2_{i}=mh\text{\_}attention\left(Input2_{i}\right),$$ (8)

$$F\text{\_}con_{i}=contenate\left(F\text{\_}out1_{i},F\text{\_}out2_{i},Input1_{i},Input2_{i}\right),$$ (9)

$$Output_{i}=dropout\left(1D\text{\_}CNN\left(F\text{\_}con_{i}\right),\;p=0.5\right).$$ (10)

## 3 Results

### 3.1 Experimental settings

The developed DCMA model was implemented in Keras, and the weights in DCMA were initialized using default values. The implementation was trained on a NVIDIA P6000 GPU. Adam optimization with an initial learning rate of 0.0004 was used to optimize the networks.

For training the model on GPU with batch input, proteins shorter than 700 AA are padded with all-zeros. Similar to the CRRNN2 experiment, the strategy of deep multi-task learning is also applied in the DCMA training period. In the inference period, only the backbone angle output is retained.

### 3.2 Evaluation metrics

To evaluate the predictive performance of protein backbone dihedral angles, the mean absolute error (MAE) (Sunghoon et al., 2011) and Pearson correlation coefficient (PCC) were used to measure the relevance between the native values and predicted ones. Here, the value of a protein dihedral angle is in the range of [ $-180^{◦}$ , $180^{◦}$ ]. Before evaluating the protein dihedral angle, the difference between the predicted value $P^{\prime }$ and the actual value *E* is usually first converted to the dihedral angle according to Equation 11 (Li et al., 2017). Then, the PCC and MAE are calculated by Equations 12, 13, respectively.

$$P=\left\{\begin{array}{c} P^{\prime },if\;\left|P^{\prime }-E\right|\leq 180^{◦}\;\\ P^{\prime }+360^{◦},if\;P^{\prime }-E\leq -180^{◦}\;\\ P^{\prime }-360^{◦},if\;P^{\prime }-E\geq 180^{◦},\; \end{array}\right.$$ (11)

where $P^{\prime }$ is the original value of the predicted dihedral angle.

$$PCC=\frac{1}{N-1}\overset{N}{\underset{i=1}{\sum }}\left(\frac{P^{\prime }-\bar{P^{\prime }}}{s_{p^{\prime }}}\right)\left(\frac{E-\bar{E}}{s_{E}}\right).$$ (12)

Among them, $\bar{P^{\prime }}$ and $\bar{E}$ are the means of $P^{\prime }$ and E, respectively, and $s_{p^{\prime }}$ and $s_{E}$ are the standard deviations of $P^{\prime }$ and E , respectively.

$$MAE=\frac{1}{N}\overset{N}{\underset{i=1}{\sum }}\left|E-P^{\prime }\right|.$$ (13)

### 3.3 Evaluation on independent test datasets

In order to better evaluate the performance of our model, we compare the DCMA model with other representative methods on public independent CASP sets. We compared the performance of DCMA with SPOT-1D, Netsurfp-2.0, DeepRIN, CRNN2, etc., on the CASP12 and CASP13 datasets, as shown in Table 1. Values in brackets are PCC, and “-” denotes data that cannot be obtained publicly. We re-implemented the model of Netsurfp-2.0 and used the same datasets and features as DCMA. DCMA achieved (19.42, 28.72) of (ϕ,ψ) on CASP12 and (19.2, 27.98) of (ϕ,ψ) on CASP13 respectively. The DCMA performance is weaker than that of SPOT-1D and OPUS-TASS and better than Netsurfp-2.0, DeepRIN, RaptorX-Angle, and SPIDER3.

**TABLE 1:** Comparison of prediction performance on the CASP12 and CASP13 datasets.

Method	ϕ		ψ	
	CASP12	CASP13	CASP12	CASP13
SPIDER3	21.12 (0.809)	-	35.67 (0.783)	-
RaptorX-Angle	20.69 (0.788)	-	31.6 (0.813)	-
DeepRIN	20.21 (0.838)	-	31.39 (0.834)	-
NetSurfP-2.0	20.0 (−)	-	31.2 (−)	-
NetSurfP- 2.0 ^a^	20.0 (0.832)	20.16 (0.838)	30.52 (0.843)	29.7 (0.847)
SPOT-1 D ^b^	18.91 (**0.84**)	18.7 (0.839)	27.46 (**0.866**)	26.64 (**0.867**)
CRRNN2	19.14 (0.842)	-	28.9 (0.853)	-
OPUS- TASS ^c^	**18.12** (−)	**17.94** (−)	**26.0** (−)	**25.95** (−)
DCMA	19.42 (0.837)	19.2 (**0.846**)	28.72 (0.857)	27.98 (0.859)

^a^ Results are generated by our reproduced experiment.

^b^ Results are from SPOT-1D’s online service.

^c^ Data are computed by their public predicted results.

Boldface numbers indicate the best performance, and “-” denotes data that cannot be obtained publicly.

The comparison between state-of-the-art models, including SPOT-1D, OPUS-TASS, OPUS-TASS2, and DCMA, is further analyzed on free modeling targets in Table 2, 3. The prediction results of OPUS-TASS2 based on sequence features are compared. The sequence features of OPUS-TASS2 are 20-D PSSM, 30-D HMM, 7-D physicochemical properties, and 19-D PSP19 features, which are denoted as “OPUS-TASS2 (76D).” Predicting results on the CASP12-FM dataset show that the performance of SPOT-1D and OPUS-TASS is similar and better than others. The performance of OPUS-TASS2 is best on the CASP-FM (56) dataset. DCMA achieved better prediction performance on the more difficult CASP13-FM dataset. On the most difficult CASP14-FM dataset, the prediction performance of OPUS-TASS2 and DCMA is comparable and better than other predictors.

**TABLE 2:** Results of predicting ϕ by different predictors on free modeling targets.

Predictor	CASP-FM(56)	CASP12-FM	CASP13-FM	CASP14-FM
NetSurfP- 2.0 ^a^	20.55 (0.835)	22.66 (0.826)	22.68 (0.8)	22.32 (0.789)
SPOT-1D-Single	-	25.43 (−)	25.13 (−)	-
SPOT-1D	19.39 (−)	22.22 (0.827)^b^	**20.8** (0.808)^b^	23.19 (−)
OPUS-TASS	18.85 (−)	**21.9** (−)^c^	22.15 (−)^c^	21.91 (−)
OPUS-TASS2 (76D)	**18.58 (−)**	-	-	**21.79(−)**
DCMA	19.44 (0.847)	22.19 (0.831)	20.84 (0.817)	21.8 (0.794)

^a^ Results are generated by our reproduced experiment.

^b^ Results are from SPOT-1D’s online service.

^c^ The results are obtained locally using the OPUS-TASS standalone package.

Boldface numbers indicate the best performance, and “-” denotes data that cannot be obtained publicly.

**TABLE 3:** Results of predicting ψ by different predictors on free modeling targets.

Predictor	CASP-FM(56)	CASP12-FM	CASP13-FM	CASP14-FM
NetSurfP-2.0^a^	32.1 (0.832)	36.3 (0.816)	34.55 (0.821)	41.1 (0.759)
SPOT-1D-Single	–	43.46 (−)	45.23 (−)	–
SPOT-1D	30.1 (−)	34.71 (0.83)^b^	29.96 (0.853)^b^	43.98 (−)
OPUS-TASS	28 (−)	**33.63(−)** ^c^	32.4 (−)^c^	38.93 (−)
OPUS-TASS2 (76D)	**26.91**	–	–	38.65
DCMA	29.31 (0.854)	34.66 (0.833)	**29.9(0.854)**	**38.57(0.772)**

^a^ Results are generated by our reproduced experiment.

^b^ Results are from SPOT-1D’s online service.

^c^ The results are obtained locally using the OPUS-TASS standalone package.

Boldface numbers indicate the best performance, and “-” denotes data that cannot be obtained publicly.

The latest benchmark dataset CASP15 (removing four similar sequences) was also used for evaluating the model generalization and achieved (19.78, 29.53) of MAE metrics and (0.83, 0.847) of PCC metrics on (ϕ,ψ) . For a more detailed understanding of the predicted results, the average dihedral angle prediction errors (measured by the MAE) are demonstrated on the CASP-FM(56), CASP12-FM, CASP13-FM, and CASP14-FM datasets across eight types of secondary structures, as shown in Table 4. The prediction errors of both H and E are the lowest because the secondary structures of H and E have the most samples.

**TABLE 4:** MAE of torsion angles on the 8-class secondary structures for the free modeling targets of the CASP datasets.

		H	B	E	G	I	T	S	C
CASP-FM(56)	phi	11.42	45.52	25.38	29.27	0	38.81	62.66	45.4
	psi	7.39	27.76	19.63	16.29	0	28.89	34.78	29.41
CASP12-FM	phi	8.78	27.31	20.97	16.87	0	29.87	38.16	31.68
	psi	15.03	47.53	28.74	35.95	0	41.48	61.76	51.04
CASP13-FM	phi	7.34	25.48	20.38	13.13	0	24.75	40.80	31.62
	psi	10.70	41.58	21.17	30.30	0	32.85	69.57	46.98
CASP14-FM	phi	8.44	28.98	22.57	21.40	0	36.13	36.72	29.81
	psi	16.53	38.29	32.74	55.29	0	55.67	69.81	56.21

For the eight class definitions: G = 3–10 helix, H = α helix, I = π helix, B = β bridge, E = extended strand, S = bend, T = h-bonded turn, and C = coil.

**FIGURE 5:**
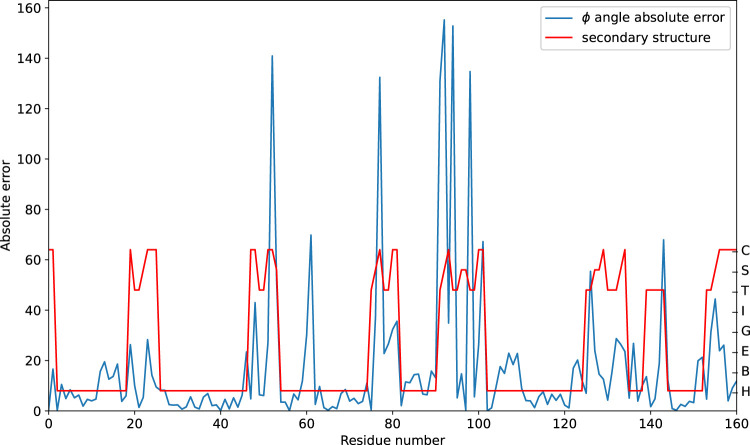
Predicting the absolute error of the ϕ angle on sequence T1039-D1. The secondary structure is linearized for visualization.

The prediction performance at the sequence level is further analyzed. The absolute error on the ϕ angle of sequence T1039-D1, selected from the CASP14-FM dataset, is visualized in Figure 5. Although the MAE at the sequence level is 16.92, the absolute errors on some residues are large. The predictive performance is acceptable on continuous secondary structure regions. When the structural state changes, prediction errors are high. We suppose that the discontinuous regions cannot supply more contextual features.

### 3.4 Ablation study

**FIGURE 4:**
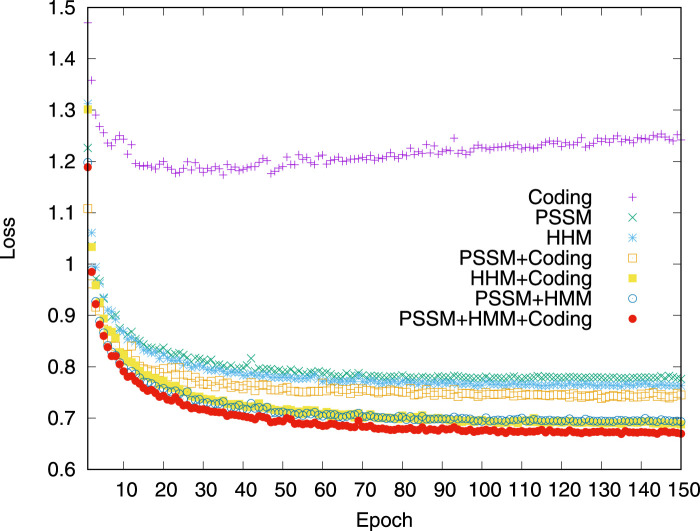
Model loss variation in the validation dataset. The comparison of the dihedral angle prediction performance of the iterative procedure using different input features on the validation dataset.

The impact of different groups of input features is first analyzed. The loss variation on the validation dataset is compared when the model was trained by using different combinations of input features. The experimental results are shown in Figure 4. Compared to the models trained by the input of a single feature, the features of pairwise combinations are more efficient in reducing losses. When three groups of features, PSSM profile, HHM profile, and residue coding, are combined, the effect of reducing loss is best.

We further analyzed the DCMA model structure. The results of the ablation experiment are shown in Table 5, where values in the cell and the bracket are the MAEs of ( ϕ , ψ ). Assuming the same other hyper-parameters, the performance of four, five, and six stacked I2A1 modules is compared. The experimental results show that the model with five stacked I2A1 modules is more effective and has fewer parameters. The influence of the multi-head attention network is also analyzed. The DCMA model removed the multi-head attention block, and the effectiveness was weakened.

**TABLE 5:** Comparison of different stacked I2A1 blocks with the same hyper-parameters.

Test dataset	Four blocks	Five blocks	Six blocks	Five blocks without attention
CASP12	19.57 (29.27)	19.42 (**28.72**)	**19.32** (28.82)	19.44 (28.99)
CASP13	18.91 (28.63)	**19.2(27.98)**	19.35 (28.31)	19.32 (28.23)
CASP12-FM	22.4 (35.56)	22.19 (**34.66**)	**22.05** (35.16)	22.35 (35.19)
CASP13-FM	21.28 (31.42)	**20.84(29.9)**	22.13 (31.01)	22.37 (31.70)
CASP14-FM	21.91 (39.78)	**21.8(38.57)**	22.16 (39.83)	22.52 (39.43)

Boldface numbers indicate the best performance.

**FIGURE 6:**
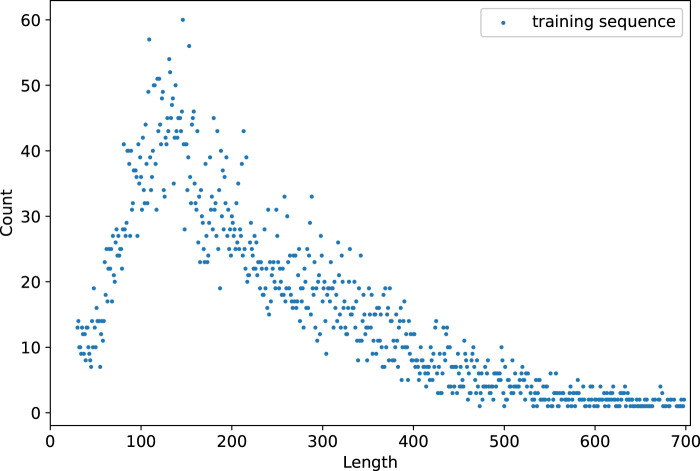
Length distributions of training and validation datasets.

The length distributions of training and validation datasets are shown in Figure 6. The data statistics show a total of 2022 sequences ranging in length from 102 to 213. In addition, there are 7,326 sequences with lengths ranging from 50 to 300. The recommended length of input sequence ranges from 50 to 300.

## 4 Conclusion

Predicting protein 3D structures is an important and challenging task. Predicting protein backbone torsion dihedral angles helps solve the problem. Heavy models are unfriendly and unsuitable for running on edge computing devices. In particular, the file size of the SPOT-1D model is larger than 10 GB. In this article, a lightweight, faster, and individual model named DCMA is proposed. The model file of DCMA is less than 50 MB. We use hybrid dilated CNN and multi-head attention to design a new deep network structure, I2A1, that substitutes for RNN. The I2A1 block balanced the model generalization and computational efficiency well. Thus, our model mechanism can be applied to predicting various other protein attributes as well.

In future work, input residues will be characterized with more structural information, including physicochemical properties and protein domains (Guo et al., 2003; Yu et al., 2023), to improve the performance of discontinuous or isolated secondary structures. Although DCMA is more lightweight and faster, its input still relies on multi-sequence alignment information such as a PSSM profile. A single-sequence-based method that did not use evolutionary features would be more friendly.

Pre-trained protein language models (pLMs) can generate information-rich representations of sequences. Combined with sequence embedding generated by pLMs, a downstream predictor of backbone dihedral angles and other 1D structural properties can be exploited without generating multi-sequence alignment information.

## Data Availability

The original contributions presented in the study are included in the article/Supplementary Material; further inquiries can be directed to the corresponding author/s.
